# Dose-finding and efficacy confirmation trial of the superselective intra-arterial infusion of cisplatin and concomitant radiation therapy for locally advanced maxillary sinus cancer (JCOG1212): final analysis

**DOI:** 10.1007/s10147-025-02702-8

**Published:** 2025-02-06

**Authors:** Hirotaka Shinomiya, Kazuto Matsuura, Rikiya Onimaru, Akira Ohkoshi, Yuki Saito, Hiroyuki Tachibana, Kiyoto Shiga, Tsutomu Ueda, Yukinori Asada, Hirokazu Uemura, Takeshi Beppu, Akira Seto, Ryuji Yasumatsu, Mitsuhiko Nakahira, Go Omura, Takahiro Asakage, Shujiro Minami, Takashi Fujii, Yuji Hirayama, Daisuke Yoshida, Kenichi Nakamura, Keita Sasaki, Junki Mizusawa, Haruhiko Fukuda, Akihiro Homma, Head and Neck Cancer Study Group of the Japan Clinical Oncology Group (JCOG-HNCSG)

**Affiliations:** 1 Department of Otolaryngology-Head and Neck Surgery, Kobe University Graduate School of Medicine, 7-5-2 Kusunoki-Cho Chuo-Ku, Kobe, Hyogo 650-0017 Japan; 2 Department of Head and Neck Surgery, National Cancer Center Hospital East, Kashiwa, Japan; 3 Department of Radiation Oncology, Tonan Hospital, Sapporo, Japan; 4 Department of Otolaryngology, Head and Neck Surgery, Tohoku University Hospital, Sendai, Japan; 5 Department of Otolaryngology, Head and Neck Surgery, University of Tokyo Hospital, Tokyo, Japan; 6 Department of Radiation Oncology, Aichi Cancer Center Hospital, Nagoya, Japan; 7 Department of Head and Neck Surgery, Iwate Medical University, Shiwa, Japan; 8 Department of Otorhinolaryngology, Head and Neck Surgery, Hiroshima University Hospital, Hiroshima, Japan; 9 Department of Otolaryngology-Head and Neck Surgery, Miyagi Cancer Center Hospital, Sendai, Japan; 10 Department of Otolaryngology-Head and Neck Surgery, Nara Medical University, Kashihara, Japan; 11 Division of Head and Neck Surgery, Saitama Cancer Center, Saitama, Japan; 12 Department of Head and Neck Surgery, Cancer Institute Hospital, Japanese Foundation for Cancer Research, Tokyo, Japan; 13 Department of Otolaryngology-Head and Neck Surgery, Faculty of Medicine, Kindai University, Osaka, Japan; 14 Department of Head and Neck Surgery, Saitama Medical University International Medical Center, Hidaka, Japan; 15 Department of Head and Neck Surgery, National Cancer Center Hospital, Tokyo, Japan; 16 Department of Head and Neck Surgery, Institute of SCIENCE TOKYO, Tokyo, Japan; 17 Department of Otolaryngology, NHO Tokyo Medical Center, Tokyo, Japan; 18 Department of Head and Neck Surgery, Osaka International Cancer Institute, Osaka, Japan; 19 Department of Head and Neck Surgery, Hyogo Cancer Center, Akashi, Japan; 20 Department of Diagnostic and Interventional Radiology, Hokkaido University Hospital, Sapporo, Japan; 21 Japan Clinical Oncology Group Data Center/Operations Office, National Cancer Center Hospital, Tokyo, Japan; 22 Department of Otolaryngology-Head and Neck Surgery, Hokkaido University Hospital, Sapporo, Japan

**Keywords:** Maxillary sinus cancers, Intra-arterial infusion, RADPLAT, Late adverse event, Head and neck cancer

## Abstract

**Background:**

JCOG1212 is a dose-finding and efficacy confirmatory study of concurrent superselective intra-arterial infusion of cisplatin and radiotherapy (RADPLAT) for locally advanced primary squamous cell carcinoma of the maxillary sinus (cT4a,bN0M0). In this study, we report the results of the final analysis of the efficacy confirmation phase for the T4a cohort with 5-year follow-up data to evaluate the late adverse events and long-term efficacy.

**Methods:**

Based on the results of the dose-finding phase, the efficacy confirmation phase consisted of seven weekly intra-arterial infusions of cisplatin 100 mg/m^2^ combined with radiotherapy (70 Gy). The 5-year prognosis and late adverse events were evaluated.

**Results:**

Between April 2014 and August 2018, 64 patients were included in the analysis (one ineligible patient was excluded); 31 patients were treated with three-dimensional conformal radiation therapy (3D-CRT) and 33 with intensity modulated radiation therapy (IMRT). The 5-year overall survival, event-free survival, and local event-free survival was 71.9, 54.7, and 57.5%, respectively. In terms of late adverse events, grade 3 or higher non-hematologic toxicity was observed in 42.9% of 63 patients (retinopathy: 12, cataract: 10, osteonecrosis of mandible: 4, etc.). Grade 3 and 4 cataracts of affected side appeared in 22.6% (7/31) of the 3D-CRT group compared to 3.1% (1/32) in the IMRT group. Twenty-one patients had died, with 15 from the primary disease, 5 from other causes, and 1 from treatment-related cause.

**Conclusion:**

The prognosis of RADPLAT was favorable after 5-year follow-up with acceptable late adverse events and low proportion of treatment related death.

## Introduction

Maxillary sinus squamous cell carcinoma (MS-SCC) is often detected at an advanced stage because of the lack of symptoms in early-stage cases [1, 2]. Surgery for locally advanced maxillary sinus squamous cell carcinoma (LA-MS-SCC) leads to changes in appearance and functional impairment related to oral intake and articulation [3]. In some cases, it is necessary to sacrifice the affected eye. Intra-venous chemoradiotherapy has been performed to preserve appearance and function, but its effectiveness has been limited, due in part to the large tumour volume of LA-MS-SCC [2, 4].

Robbins et al. developed a therapy in which high-dose cisplatin is injected arterially through the arteries feeding the tumor, and neutralized cisplatin intravenously with sodium thiosulfate [5, 6]. They reported that the arterial infusion chemoradiotherapy for head and neck cancer, named RADPLAT, showed good oncological results [7]. Subsequently, good clinical outcomes were reported with RADPLAT for LA-MS-SCC [8–10], and it was expected afford a promising function-preserving treatment. However, a randomized trial in the Netherlands comparing arterial and intravenous infusion reported no significant difference in locoregional control or overall survival (OS) between the two arms [11]. This trial enrolled patients with head and neck cancers other than maxillary cancer, and included many bilateral cases, which may have affected the results by including patients who were less likely to benefit from arterial infusion chemoradiotherapy. Therefore, we conducted a dose-finding and efficacy confirmation trial of the superselective intra-arterial infusion of cisplatin and concomitant radiotherapy for LA-MS-SCC (JCOG1212) [12].

We have already reported the primary analysis of the efficacy confirmation phase in the T4a cohort [13]. We demonstrated that RADPLAT showed favorable results for patients with T4aN0M0 disease (3-year OS: 82.8% (90% CI, 73.4–89.2%)) compared with the historical control for 3-year OS based on surgery (80%). There were no unacceptable acute complications except for one death from a treatment-related pulmonary embolus. Based on this, RADPLAT, as well as surgery, can be regarded as a possible treatment option for these patients through the primary study. However, this treatment involves localized radiation therapy (70 Gy) and up to seven arterial infusions of cisplatin, which may increase late complications such as radiation osteonecrosis and visual function-related disorders, as well as treatment-related death. This report presents the final analysis from the updated data with 5-year follow-up and late adverse events.

## Patients and methods

### Eligibility

Eligibility criteria in the T4a cohort a, which were reported previously [13], are summarized below: primary lesion located at the maxillary sinus (MS); histologically proven squamous cell carcinoma (SCC); clinical stage T4aN0M0; no severe carotid stenosis as evaluated by ultrasonography; aged between 20 and 75 years; Eastern Cooperative Oncology Group performance status (ECOG PS) of 0 or 1; no prior therapy for maxillary sinus cancer; no prior radiotherapy to the head and neck or brain; no prior chemotherapy for any other malignancies; sufficient organ function; the ability to reach the external carotid arteries from the femoral artery with a catheter; and satisfying the normal tissue radiation dose constraints for the ipsilateral eyeball and optic nerve, spinal cord, brainstem, and chiasma. Written informed consent was obtained from all patients before registration.

### Study design

JCOG1212 is a multi-institutional, single-arm, prospective interventional study of superselective intra-arterial infusion of high-dose cisplatin with concomitant radiotherapy for patients with T4aN0M0 and T4bN0M0 LAMSC conducted in 18 institutions [12]. The trial consisted of a dose-finding phase [14] and an efficacy confirmation phase [13]. It was registered with the UMIN Clinical Trials Registry under trial number UMIN000013706 and the Japan Registry of Clinical Trials (Number: jRCTs031180004).

The dose-finding phase was designed to determine the recommended number of cycles through the study of patients with either T4aN0M0 or T4bN0M0 tumors. In this phase, 100 mg/m^2^ of cisplatin was administered intra-arterially weekly for 7 weeks with concomitant radiotherapy (70 Gy/35 fractions). The results indicated that this therapy is safe and well-tolerated at 7 cycles of cisplatin, which was determined to be the recommended number of cycles for locally advanced MS-SCC [14].

In the efficacy confirmation phase, the objective is to evaluate the efficacy and safety of RADPLAT for patients with locally advanced MS-SCC. The efficacy confirmation phase is being conducted separately for patients with T4aN0M0 and T4bN0M0 MS-SCC. We have already reported the efficacy and adverse events for T4aN0M0 [13], but not for T4bN0M0.

Per-protocol disease assessment (physical examination, magnetic resonance imaging of the head and neck, and thoracoabdominal computed tomography) and adverse event data were required every 3 months for the first year, every 4 months for year 2 and 3, and then every 6 months for year 4 and 5. All patients who were enrolled in the trial were to be followed for at least 5 years, while analysis of the primary endpoint of the efficacy confirmation phase was planned to be conducted 3 years after accrual completion.

### Endpoints

The primary endpoint of the efficacy confirmation phase was the 3-year OS, and the secondary endpoints were event-free survival (EFS), local event-free survival (LEFS), clinical complete response rate, incidence of adverse events, and serious adverse events. We already reported the 3-year OS was 82.8% (90% CI, 73.4–89.2%) as the primary endpoint [13]. This report presents the 5-year OS, EFS, LEFS, and incidence of late adverse events. OS was computed from the date of registration to the time of death from any cause. EFS was computed from the date of registration to the death from any cause, any progression (including recurrence), and salvage surgery. LEFS was computed from the date of registration to the death from any cause, primary disease progression (including recurrence), and salvage surgery for the primary lesion. The clinical complete response rate was determined from the proportion of complete responses (CRs) and good partial responses (good PRs) among all eligible patients. A good PR was characterized as a secondary change unique to the post-CRT period and which was regarded as a residual scar but not as a residual tumor. A good PR in this study was defined as lesions ≤10 mm in size or those not enhanced on contrast-enhanced MRI. This criterion was used in JCOG0706 [15] by the JCOG Head and Neck Cancer Study Group, and was applied in this study as well. Toxicities were evaluated according to the Common Toxicity Criteria for Adverse Events version 4.0.

### Treatment methods

The previously reported treatment methods [13] are summarized below. The protocol treatment consisted of weekly superselective intra-arterial infusion of cisplatin with concomitant radiotherapy and salvage surgery where necessary.

### Chemotherapy

One hundred mg/m^2^ of cisplatin was administered intra-arterially weekly for 7 weeks. At the same time, sodium thiosulfate was administered at a dose of 20 g/m^2^ intravenously to neutralize the cisplatin. Seven cycles, which was determined to be the recommended number of cycles in the dose-finding phase [14], was applied in the efficacy confirmation phase. To control the quality of the interventional technique, central review of photographs and movies in arbitrarily selected patients was performed at a semiannual investigators’ meeting. All interventional procedures were performed or directly supervised by interventional radiologists certified by the study chair.

### Radiation therapy

Three-dimensional conformal radiation therapy (3D-CRT) or intensity-modulated radiation therapy (IMRT) was chosen at institutional discretion (this trial began in April 2014 and IMRT became available after March 2016). For quality control and assurance of radiation therapy, compliance with protocol-specified radiation therapy planning was examined for all enrolled patients at completion of radiation therapy. Radiation therapy was administered with high-energy photons of 4–10 MV X-rays to a total dose of 70 Gy in 2 Gy fractions five times weekly. The protocol specified that any reduction in the PTV margin solely for the reason of lowering the dose to the affected eye was not acceptable, based on the idea that the risk of visual impairment in this patient group is deemed acceptable.

### Statistical analysis

OS, EFS, LEFS were calculated by the Kaplan–Meier method. The confidence intervals for the complete response rate were calculated using the exact methods. Hazard ratios were computed using a univariable Cox proportional hazards model to compare the efficacy between the IMRT group and the 3D-CRT group. All analyses were performed using SAS version 9.4.

## Results

### Patient characteristics

From April 2014 to August 2018, 65 patients were registered in the T4a cohort from 18 institutions, consisting of 54 males and 11 females with a median age of 64 years (range, 40–78 years) and an ECOG performance status of 0/1 (58/7) (Table 1). After the exclusion of one ineligible patient who did not fulfil the inclusion criteria due to moderate or greater stenosis of the common and internal carotid arteries, 64 patients were included in the primary analysis of efficacy and safety. Thirty-one patients received 3D-CRT and 32 received IMRT.

**Table 1:** Baseline characteristics of all enrolled patients (N = 65)

Characteristic	Value
Age, y	
Median	64
Range	40–78
Sex	
Male	54
Female	11
ECOG performance status	
0	58
1	7
T classification	
T4a	65
Histology	
Well differentiated	7
Moderately differentiated	16
Poorly differentiated	13
Unknown	28
Missing	1
Radiation method (N = 64※)	
3DRT	31
IMRT	33

*ECOG* Eastern Cooperative Oncology Group, *3DRT* three-dimensional conformal radiation therapy, *IMRT* intensity modulated radiation therapy

※One patient who was ineligible after enrollment was excluded

### Efficacy

**Fig. 1:**
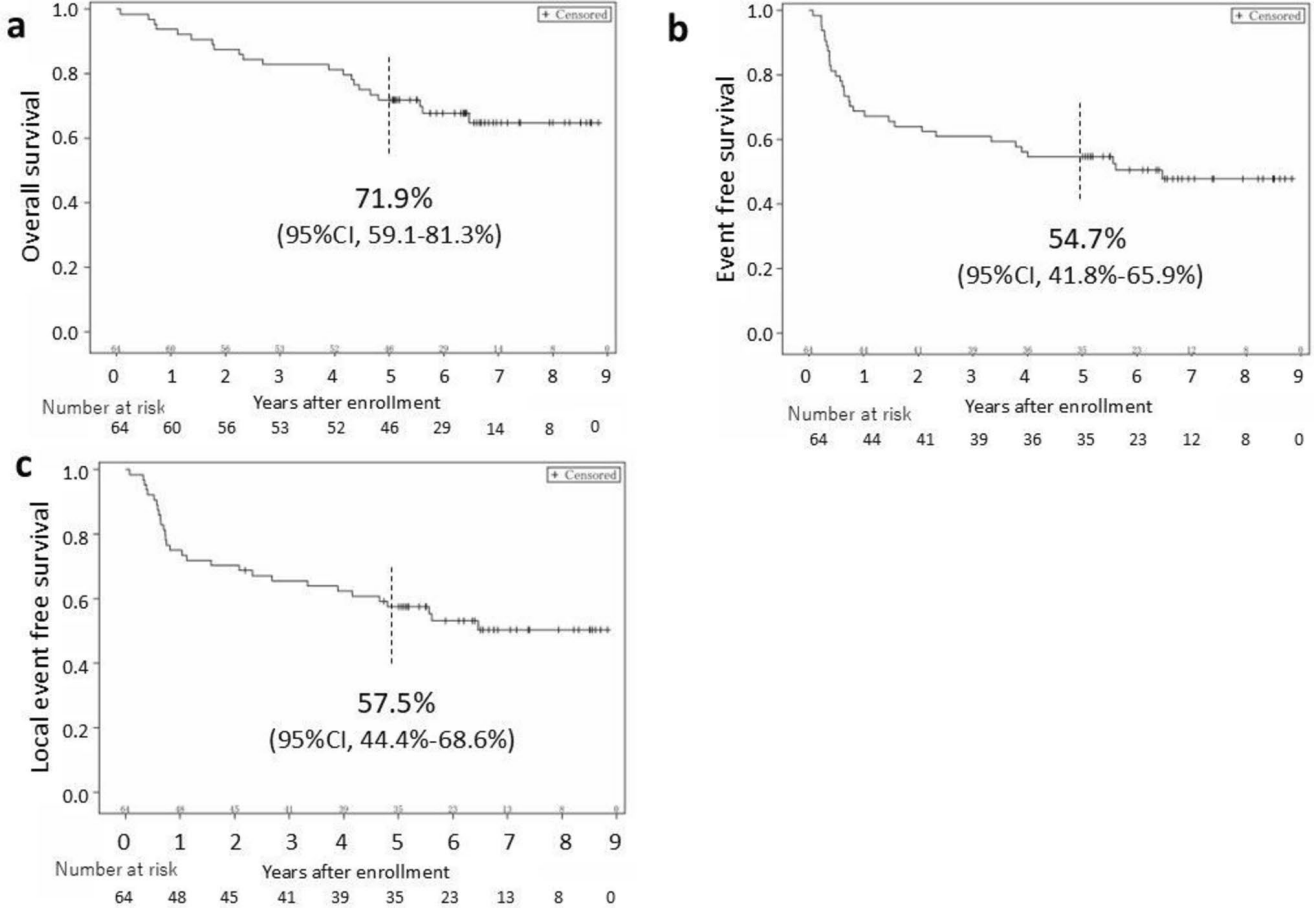
**a** The Kaplan–Meier curve for overall survival (OS) for all eligible patients. The symbols indicate censored observations. The 5-year OS was 71.9% (95% CI, 59.1–81.3%). **b** The Kaplan–Meier curve for event-free survival (EFS) for all eligible patients. **c** The Kaplan–Meier curve for local EFS for all eligible patients

The median follow-up period was 5.8 years in all eligible patients and 6.6 years in the 43 survivors. The 5-year OS was 71.9% (95% CI, 59.1–81.3%) (Fig. 1a). The 5-year EFS and 5-year LEFS were 54.7% (95% CI, 41.8–65.9%) and 57.5% (95% CI, 44.4–68.6%), respectively (Fig. 1b, c). The 5-year OS of the IMRT group was 75.8% and the 3D-CRT group was 67.7% (HR:1.195 (95% CI: 0.504–2.833)). The 5-year LEFS of the IMRT group was 59.9% and the 3D-CRT group was 54.8% (HR: 1.008 (95% CI: 0.490–2.073)).

Regarding the cause of death, 15 patients died with disease, while there was one treatment-related death and 5 deaths due to other causes; for example, other malignancies, unrelated cerebral hemorrhage, trauma, and sudden death of unknown cause. Compared to the results presented in the previously published primary report [13], there was an increase of three deaths caused by the disease and an increase of four deaths from other causes.

### Late adverse events

Median total dose and duration of radiotherapy was 70 Gy (interquartile range: 70–70 Gy) and 51 days (interquartile range: 50–52 days), respectively, with the median number of cycles of cisplatin being 7 (interquartile range: 6.5–7 cycles). One treatment-related death due to a pulmonary embolism was reported. Late adverse events are shown in Table 2 according to each radiation technique. In terms of late adverse events (≥grade 3), retinopathy in 12 (19.0%), cataract in 10 (15.8%) and osteonecrosis of the mandible in 4 (6.3%) patients were observed. Two patients had Grade 4 corneal ulceration. These patients received a maximum dose of 73.6 and 73.8 Gy, respectively, to the eyeball on the affected side, and both were treated with IMRT. Brain necrosis occurred in three cases, but all were Grade 1 with no symptoms. Grade 3 or higher osteonecrosis only increased from 2 to 4 cases from the previous report, and Grade 3 or higher visual function-related complications (cataract, corneal ulcer, retinopathy, and glaucoma) on the affected side increased by only 1 case from the previous report. Grade 3 and 4 cataracts of affected side appeared in 22.6% (7/31) of patients in the 3D-CRT group compared to 3.1% (1/32) in the IMRT group. Further, Grade 3 and 4 retinopathy was observed in 25.8% (8/31) of patients in the 3D-CRT group compared to 12.5% (4/32) in the IMRT group.

**Table 2:** Late adverse events according to radiation technique

Radiation technique	3D-CRT (n = 31)					IMRT (n = 32)					*p* value
Late Adverse Event	G1	G2	G3	G4	%G3–4	G1	G2	G3	G4	%G3–4	
Hearing impairment	2	3	0	0	0	6	1	0	0	0	1
Peripheral sensory neuropathy	1	0	0	0	0	1	2	0	0	0	1
Fatigue	1	1	0	–	0	0	2	1	–	3.1	0.49
Osteonecrosis of mandible	1	2	2	0	6.5	2	0	2	0	6.3	1
Trismus	3	1	0	–	0	6	1	1	–	3.1	0.49
Brain necrosis	2	0	0	0	0	1	0	0	0	0	1
Stroke	0	0	0	0	0	1	0	0	0	0	1
Facial nerve disorder	1	0	0	–	0	1	0	0	–	0	1
Watering eyes*	14	4	0	–	0	12	1	1	–	3.1	0.49
Cataract*	0	1	6	1	22.6	3	2	1	0	3.1	0.026
Corneal ulcer*	–	5	1	0	3.2	–	0	0	2	6.3	1
Retinopathy*	2	2	5	3	25.8	0	0	3	0	9.4	0.11
Glaucoma*	0	0	2	1	9.7	1	0	0	0	0	0.11
Periorbital infection*	–	1	0	0	0	–	1	0	0	0	1
Cataract**	2	0	2	0	6.5	0	0	0	0	0	0.49

^*^Affected side, **healthy side

### Pattern of relapse and salvage surgery

**Fig. 2:**
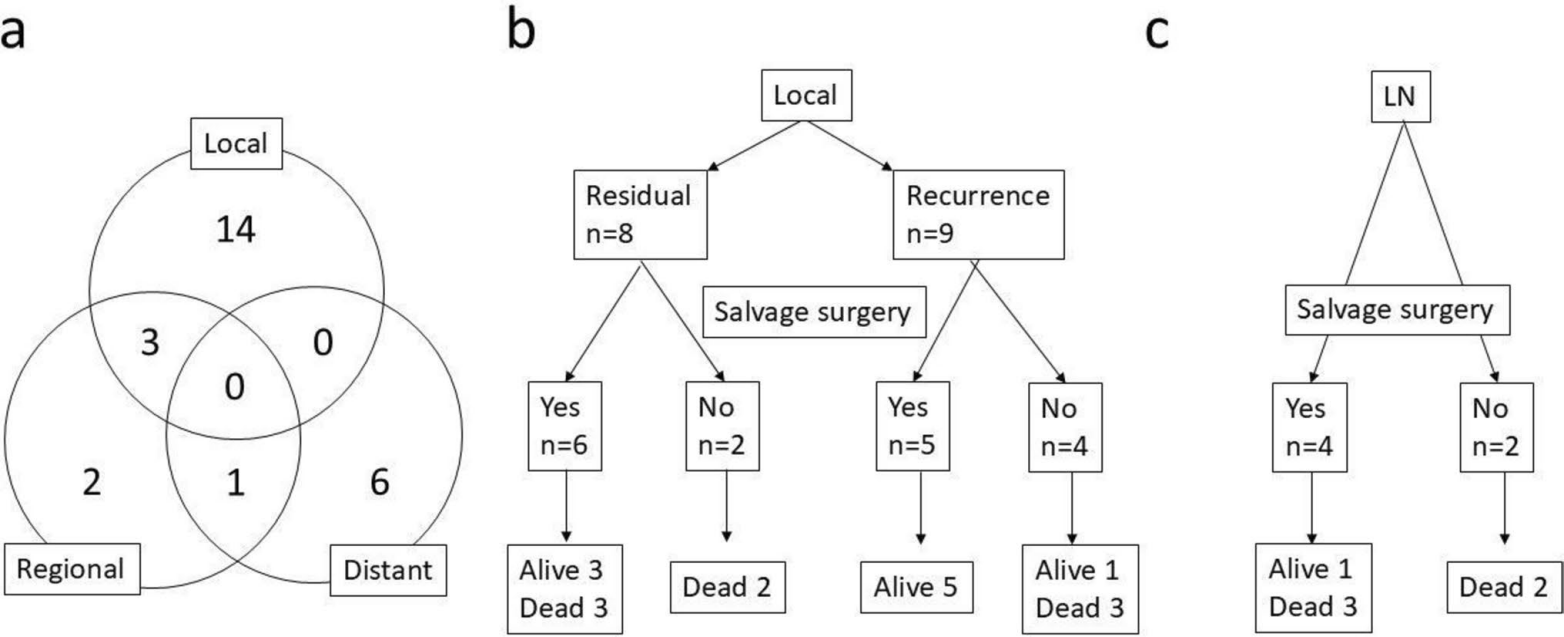
**a** Pattern of recurrence and **b** the timing and outcomes for patients with residual/recurrent primary disease and **c** for those with recurrent neck disease

Twenty-six patients had residual or recurrent diseases as follows: the primary site was involved in 17, regional lymph nodes involved in 6, and distant metastasis in 7 cases, with some overlap among patients (Fig. 2a). In terms of the primary site, 6 of the 8 patients with residual disease received salvage surgery, with 3 of them alive and 3 dead to date. On the other hand, 5 of the 9 patients with recurrent disease received salvage surgery. All patients who received salvage surgery for locally recurrent disease are alive, but 3 of the 4 patients without salvage surgery died of disease (Fig. 2b). Six patients had regional lymph node recurrence, with 4 of them undergoing neck dissection. As a result, 1 of the 4 patients who received surgery is alive to date, with the remaining 5 patients dead (Fig. 2c).

## Discussion

We demonstrated that RADPLAT showed favorable results for patients with T4aN0M0 MS-SCCs with no significant increase in late adverse events. Standard therapy for LA-MS-SCC is surgical resection and postoperative radiotherapy; however, the 5-year OS is poor at 36–52%, with the most frequent cause of death being local recurrence [2, 16]. Surgical resection along with free flap reconstruction has been utilized as the standard treatment, but this approach is associated with postoperative esthetic issues, trismus, and vision disturbances.

In recent years, the efficacy of RADPLAT, a form of curative IA-CRT administered via the Seldinger method, has been reported for LA-MS-SCC [8–10]. These reports have shown high 5-year survival rates ranging from 69.3 to 78.4%, primarily for T4 cases. IA-CRT has the advantage of organ preservation and a therapeutic effect that is similar to that of surgical resection. We have already reported that RADPLAT showed favorable results for patients with T4aN0M0 compared with the historical control for 3-year OS based on surgery in the primary analysis [13]. According to this final analysis, the 5-year OS was good, with no considerable survival loss occurring over a longer period of time. Moreover, the added deaths were caused by other causes in 4 patients, with only 3 caused by the disease. Although local recurrence was the most common recurrence pattern, most of them were salvaged by surgery. On the other hand, most cases with distant metastases or regional lymph node metastases could not be salvaged. The recurrence pattern is also very different from that observed in the Dutch randomized trial that compared IA-CRT and IV-CRT for inoperable SCC of the oropharynx, oral cavity, or hypopharynx [11]. In the Dutch trial, distant metastatic recurrence was observed in 65 cases compared to 57 cases of local recurrence [17]. The Dutch trial focused on oral and pharyngeal cancers, which are more prone to distant metastasis compared to MS-SCC. On the other hand, in our study, the number of distant metastasis was low compared to that of local recurrence. As distant metastasis is relatively unlikely to occur in MS-SCC, we speculate that RADPLAT contributed to survival by increasing the intensity of local treatment.

Early diagnosis of local recurrence is the first step to improving survival. When salvage surgery can be performed, a high salvage proportion is achieved. Although it is difficult to distinguish between posttreatment scarring and recurrent lesions on imaging studies, early diagnosis is achieved by combining multiple modalities. Actually, in this study, most of the cases with local recurrence could be treated with salvage surgery. Long-term survival was achieved in cases in which salvage surgery could be performed. We believe that the high proportion of salvage for local recurrence contributes to the improved survival. Prophylactic radiation therapy to regional lymph nodes was not performed in this study. Late regional metastasis was observed in a few case, and many of these cases were not salvageable. Thus, early diagnosis and salvage treatment of delayed lymph nodes remain an issue.

Acute adverse events were already reported to be equally frequent with the standard 3-weekly cisplatin RT regimen, although high-dose cisplatin was administered [13]. This is thought to have been due to the neutralization of cisplatin by sodium thiosulfate. We were concerned about increased late adverse events due to the 70 Gy radiation therapy and an average of 7 doses of 100 mg/m^2^ cisplatin locally, with particular concern about increased radiation osteonecrosis, brain necrosis, and visual function-related complications. Fortunately, however, these late complications showed little increase since the previous report. Shokri et al. reported that in their review of 80 patients of radiogenic maxillary osteonecrosis, 74% of them occurred within 3 years of treatment, with tooth extraction being a factor in half of them [18]. Similar to that report, we observed no significant increase in cases of osteonecrosis after 3 years of treatment. In many cases, the affected eye is in close proximity to the tumor, and removal of the eyeball is required by surgical treatment. Therefore, high doses of radiation are administered to the affected eye, and the treatment allows for some loss of vision in that eye. However, the incidence of cataract, corneal ulcer, retinopathy, and glaucoma in Grade 3 and Grade 4 patients was less than 20%, indicating that visual function was preserved at a better-than-expected proportion. Actually, Ashraf et al. [2] reported that 34 of 63 cases of T4 maxillary carcinoma treated with surgery were required with orbital content removal. One advantage of RADPLAT over surgery is that it prevents facial deformities, including preservation of the eyeball, and may also provide additional benefits in terms of the preservation of visual function. In particular, significantly fewer cases of cataract were observed on the affected side in the group using IMRT than in the group using 3D-RT, suggesting that the use of IMRT may contribute to visual function preservation by limiting the dose to the eye. Konishi et al. [19] similarly performed RADPLAT in 58 patients with advanced maxillary carcinoma and reported that IMRT was associated with fewer visual complications than observed for 3D-RT. Further study of the relationship between ocular dose and preservation of visual function is warranted. On the other hand, there was no difference in OS and LEFS between the IMRT and 3D-CRT groups.

This study had several limitations. First, the study included only cases with T4N0 disease among advanced maxillary carcinomas and did not present information on cases with lymph node metastases. Moreover, we cannot present results for T4b at this time as the data will be analyzed separately from T4a due to different endpoints. Second, this was a single-arm study as MS-SCC is a rare disease and it was impossible to recruit patients for a randomized trial comparing surgical interventions such as total maxillectomy, often with orbital content removal. As we did not directly compare surgical treatment with RADPLAT, no definitive conclusions of treatment superiority or inferiority can be drawn. Rather, a comparison of 3D-RT and IMRT is being made. The number of centers performing IMRT increased in the latter half of the study, and the possibility that the timing of treatment may have influenced the results cannot be ruled out.

**In conclusion,** RADPLAT for advanced maxillary sinus carcinoma T4aN0M0 showed a favorable oncologic response and no considerable increase in late adverse events, suggesting that IMRT may reduce visual dysfunction. RADPLAT is a promising treatment for advanced maxillary sinus carcinoma.
